# Specific and segregated changes to the functional connectome evoked by the processing of emotional faces: A task-based connectome study

**DOI:** 10.1038/s41598-020-61522-0

**Published:** 2020-03-16

**Authors:** Sebastian Markett, Philippe Jawinski, Peter Kirsch, Martin F. Gerchen

**Affiliations:** 1 Humboldt Universität zu Berlin, Berlin, Germany; 2 Central Institute of Mental Health, University of Heidelberg/Medical Faculty Mannheim, Mannheim, Germany; 3 Bernstein Center for Computational Neuroscience Heidelberg/Mannheim, Mannheim, Germany

**Keywords:** Amygdala, Human behaviour

## Abstract

The functional connectome is organized into several separable intrinsic connectivity networks (ICNs) that are thought to be the building blocks of the mind. However, it is currently not well understood how these networks are engaged by emotionally salient information, and how such engagement fits into emotion theories. The current study assessed how ICNs respond during the processing of angry and fearful faces in a large sample (N = 843) and examined how connectivity changes relate to the ICNs. All ICNs were modulated by emotional faces and showed functional interactions, a finding which is in line with the “theory of constructed emotions” that assumes that basic emotion do not arise from separable ICNs but from their interplay. We further identified a set of brain regions whose connectivity changes during the tasks suggest a special role as “affective hubs” in the brain. While hubs were located in all ICNs, we observed high selectivity for the amygdala within the subcortical network, a finding which also fits into “primary emotion” theory. The topology of hubs corresponded closely to a set of brain regions that has been implicated in anxiety disorders, pointing towards a clinical relevance of the present findings. The present data are the most comprehensive mapping of connectome-wide changes in functionally connectivity evoked by an affective processing task thus far and support two competing views on how emotions are represented in the brain, suggesting that the connectome paradigm might help with unifying the two ideas.

## Introduction

The human connectome is the abstraction of the intricate network that is our brain^1–3^. Functional neuroimaging techniques can be applied to study temporal dependencies of hemodynamic activity across the connectome in order to infer functional interactions between brain regions^4^. A key finding of functional connectomics is that hemodynamic activity synchronizes itself into several large scale network modules. These modules have been first described in the resting-state, a condition characterized by the absence of external stimulation, and have therefore been labeled intrinsic connectivity networks^5–8^. Intrinsic connectivity networks (ICNs) delineate along structural as well as functional boundaries in the brain and include sensory (e.g. visual), effector (e.g. sensory-motor), and association networks with assumed implications for higher order functions such as attention, emotion, salience processing, and executive control^7,9,10^. The brain network’s organization into separable ICNs extends to task states. Topological organization of co-activations across different tasks follows closely the topology of ICNs^11^ and task-evoked activity changes can be predicted from the topology of ICNs on the level of single participants^12^. Furthermore, the brain’s intrinsic architecture has been shown to persist across different task states and change only in a subtle, yet specific way once the brain engages in a cognitive or affective task^13^.

The role of ICNs in cognitive and affective functioning is a matter of ongoing research^14,15^. With regard to affective processing, it has been noted that there is no one to one correspondence between single ICNs and single basic emotions^16^. Even though basic emotions such as anxiety, anger, disgust, sadness, and happiness show unique and separable activation profiles in the brain^17^, the activation foci do not specifically fall into separable ICNs. This has been interpreted as support for the *theory of constructed emotions* that basic emotion do not arise from separable ICNs but are constructed by the interplay of several, domain-general ICNs^18,19^. With respect to anxiety, it has been hypothesized that the salience network, the default mode network, and ventral and dorsal fronto-parietal networks are engaged in healthy processing of anxiety-related information^20^ The interplay of these networks is however not only relevant for the anxiety response in healthy individuals. It has also been suggested that dysfunctional network interactions are involved in elevated levels of trait anxiety that may result in anxiety disorders at the extreme end of individual differences^20^.

The idea that the neural representation of emotions in the brain cannot be pinpointed to any particular circuit but is distributed across multiple systems as positioned by the theory of constructed emotions is in stark contrast to the traditional perspective of basic emotion^21–23^ and affective neuroscience theory^24,25^. These views conceptualize emotions as distinct and innate entities that can be localized in distinct subcortical circuitry. At present, both accounts stand against each other but a unification would be desirable^15,25^. A precise mapping of functional connectivity changes during affective processing across the entire connectome and their relationship to the intrinsic connectivity architecture of the brain would be a valuable step towards this goal.

The most widely applied paradigm to study functional activation changes in response to a task utilizes the subtraction method in which activation in an experimental condition of interest is compared to a control condition that matches the experimental condition in domain-general aspects such as sensory or motor demands. In this framework, task-evoked connectivity changes can be assessed by psychophysiological interactions (PPIs). PPIs are specialized forms of moderated multiple regression analysis that include an interaction term between a brain area’s physiological activation time course and temporal events in a psychological experiment^26^. When combined with a whole brain parcellation scheme, PPI analysis can be used to map functional connectivity changes across the entire functional connectome during different stages of a task^27^. In the present study, we apply the generalized form of PPI analysis^28^ to map functional connectivity changes in a popular affective processing task that requires participants to match face stimuli with fear and anger expressions^29^. We combine this analysis with information on the topology of ICNs which we derived from the same participants’ resting-state data. Both analyses were based on the same whole-brain parcellation scheme in order to describe within- and between network connectivity changes and to provide a comprehensive map of functional network interactions evoked by the processing of emotional faces.

Our analyses are intended to answer the following questions. First, we ask how different ICNs activate and deactivate during emotional processing. Second, we ask how functional connectivity within and between ICNs changes in response to emotional processing. Thirdly, we ask which brain regions change their connectivity in response to the processing of emotional faces and how the topology of such brain regions relates to ICNs. Lastly, we ask how task-evoked changes in functional connectivity relate to published findings on structural and functional alterations in patient populations.

## Methods

### Data and participants

All imaging data were provided by the Human Connectome Project, WU-Minn consortium^30^ (HCP, www.humanconnectome.org,). The sample included all participants from the HCP S900 release (December 2015) with complete emotion processing task data (N = 843, n = 469 female, n = 373 male, n = 1 unknown, mean age M = 28.79, SD = 3.69, range: 22–37 years). Participants were free of neurodevelopmental, psychiatric, and neurological disorders. For a detailed list of inclusion and exclusion criteria we refer to the HCP project overview article^30^.

### Task

The HCP’s emotion processing task was adapted from Hariri *et al*. (2002). Angry and fearful faces were presented in 21 s blocks, interleaved with blocks presenting geometrical shapes as control condition (six trials per block, trial duration 2 s, intertrial interval 1 s, 3 s cue at the beginning of each block indicating whether faces or shapes should be attended). Subsequent blocks were spaced with 8 s fixation periods. Participants were asked to indicate which of two faces (emotion condition) or which of two shapes (control condition) presented at the screen’s bottom matched the face (or shape) at the top of the screen. Two sessions with 176 functional volumes and a total length of 2.11 minutes each were administered. Each session included three face blocks and three shape blocks. Time to repeat (TR) was 0.72 seconds.

### Whole brain parcellation

The parcellation scheme was taken from Shen *et al*. (2013) who provide a sub-cortical and cortical grey matter parcellation optimized for functional homogeneity. The most detailed version of the atlas with 278 regions of interest (ROI) was used.

### Resting-state data

We included resting-state data from all participants with available emotion processing data (N = 843). We used resting-state data that were recorded on the same day as the task data. Two runs with opposing encoding directions (left to right and right to left) were used. Each run included 1200 time points with an acquisition time of 0.72 s per functional volume, resulting in 28.8 minutes of resting state data per participant. HCP’s resting state data are low motion data with a mean frame wise displacement of 0.162 (range 0.073–0.339)^31^. Preprocessing included all stages from HCP’s minimal preprocessing pipeline^32^, including linear regression of 24 head motion parameters and additional denoising with ICA-Fix to account for additional noise including head motion. Functional volumes were then spatially smoothed (4 mm FWHM) and bandpass-filtered (0.01–0.1 hz). Prior to analysis, scans from the two encoding directions (LR and RL) were concatenated.

Mean BOLD time series were extracted from all 278 regions of the Shen-atlas for each participant and a 278 × 278 adjacency matrix was set up for each participant based on r-to-z-transformed partial correlations. Partial correlations were computed by inverting the covariance matrix after applying LeDoit-Wolff shrinkage (L2-Regularization)^33^. Functional network detection was achieved by a consensus clustering approach as detailed in Bertolero *et al*. (2015). A consensus matrix was derived for each participant after proportionally thresholding the z-transformed partial correlation matrix at a cost of 0.035 and by running the Infomap cluster detection algorithm with 1,000 iterations on the thresholded matrix^34^. The resulting consensus matrices were averaged together across all participants and proportionally thresholded at 0.07. Then, the Infomap algorithm was applied to the thresholded group consensus matrix with 5,000 iterations. This resulted in twelve resting-state modules. Five nodes were not classified in any of the twelve modules and were discarded from further analysis^35^.

### Task data

Preprocessed volume based images were smoothed with a Gaussian kernel with 4 mm full width at half maximum. General linear models were estimated in SPM8 (http://www.fil.ion.ucl.ac.uk/spm) by convolving the experimental regressors (fear and neutral) and six movement regressors and their first temporal derivatives with a canonical hemodynamic response function (HRF) and by applying a highpass-filter of 200 s. Linearly weighted contrast images (emotion vs. neutral) were submitted to a random effect second level analysis. The resulting t-map was thresholded at p < 0.05, corrected for the family wise error rate. Only voxels exceeding an extend threshold of k > 20 voxels are reported. We identified 23 participants with insufficient whole-brain coverage in the functional task runs. These participants were excluded from the analysis, which resulted in a final sample of N = 820 for all task analyses.

### Task activation of resting-state networks

We extracted mean parameter estimates from all participants for each ICN. We used Bonferroni-adjusted one-sample t-tests to assess whether each ICN was modulated by the task and whether it got activated or deactivated.

### Whole brain psychophysiological interactions

For whole-brain PPI analyses we adopted gPPI data processing functions from SPM8 and the gPPI toolbox (http://www.nitrc.org/projects/gppi) on the level of brain regions^27^. For each participant and session, mean time-courses were extracted from the whole-brain parcellation scheme, temporally filtered (200 s highpass filter), pre-withened to account for auto-correlations and adjusted for the F-contrast over both experimental conditions and sessions.

For each ROI from the Shen-atlas, PPIs targeting all other ROIs were calculated with general linear models that included the convolved PPI interaction terms, the seed region time course, the experimental regressors convolved with the HRF, unconvolved movement regressors and their first temporal derivatives, and a constant. Regression coefficients (beta values) of the interaction terms were saved in a full connectivity matrix between all ROIs. This procedure resulted in one matrix per participant, condition, and session.

### Difference matrix

For each participant, one 273*273 contrast matrix was created using linearly weighted contrasts between conditions and across sessions. Based on these matrices, one difference matrix was derived at the group level by retaining edges that were significantly different from zero at an edge-wide threshold of p < 0.05, Bonferroni-corrected for 74,256 single comparisons (p < 6.7335e-07). A total of 2973 edges were significantly larger and 4645 edges were significantly lower than zero. Edges larger than zero were flagged with 1 in the difference matrix, edges smaller than zero with −1, and non-significant edges with 0. Because PPIs are based on moderated regression analyses, it is not guaranteed that each connection is bi-directional. Even though we will refrain from interpreting directionality, we still based all calculations on a directed and signed difference matrix.

### Network-level analysis

We used code from the Brain Connectivity Toolbox (BCT, https://sites.google.com/site/bctnet/) for network analysis^36^. The purpose of the network-level analysis was to assess task-evoked connectivity changes at the network level. We computed within- and between network densities based on the twelve resting-state networks and the difference matrix (based on the BCT function density_dir.m). Positive and negative edges were analyzed separately, resulting in one 12*12 density matrix for positive and one 12*12 density matrix for negative edges. Significance of network densities was assessed based on a permutation approach: We randomly shuffled edges in the difference matrix (10,000 permutations) while preserving signs and the in- and out-degree distribution (based on the BCT function randmio_dir_signed.m). In each permutation, each edge was rewired ten times. Resulting p-values for positive as well as negative within- and between network densities were Bonferroni adjusted (for 12*12*2 = 288 tests). To probe each network’s involvement, we calculated its network-level nodal degree (BCT function degree_dir.m). This was done separately for positive and negative densities. We used the combined in-out degree for further analysis to avoid any interpretation of directionality. The degrees for positive and negative densities were combined (summed), which resulted in a network degree vector with twelve entries. We used a χ^2^-goodness of fit test to assess whether network-level degree distribution differed from a uniform distribution of degrees across ICNs.

### Nodal-level analysis

All nodal-level analyses were based on the difference matrix. Because most nodal measures require all positives network edges, we used the absolute difference matrix (i.e. negative edges were set to one as well). We computed hub-ness for each of the 273 nodes by computing hub-scores^37^. A brain region was considered a hub when the region scored in the top 33% on at least three out of four centrality measures: nodal degree, participation coefficient, betweenness, and nodal path length. For mathematical reasons, we first computed hub-ness separately for ingoing and outgoing connections with the respective functions from BCT and then collated the two measures into a unified hub measure: A brain region was flagged as a hub when its ingoing or outgoing connections suggested hub-ness. Because the criterion for hub-ness (top 33% in three out of four measures) is somewhat arbitrary, we complemented this analysis by k-means clustering that was applied to the four centrality measures with the k-parameter set to two (in order to identify two groups: hubs and non-hubs). K-means clustering was first applied separately to ingoing and outgoing connections. Again, a brain region was flagged as a hub when its ingoing or outgoing connections suggested hub-ness. While graph-theoretical measures such as degree (amount of connections) and participation coefficient (diversity of connections across network modules) can be straightforwardly interpreted, the biological implication of path-based measures such as nodal path length or betweenness are less intuitive, particularly when applied to functional brain networks (Fornito *et al*., 2013^38^, Bullmore & Sporns, 2009^39^). By using an aggregate measure such as hub-ness, we simply identify a subset of brain regions whose connectivity patterns suggest a high responsiveness to the task and a high relevance for the functional network while at the same time mitigating the problem of overinterpreting the biological implications of single measures. We used permutation-based testing to assess whether more or less hubs fell in each of the twelve networks than expected by chance. A null-distribution was created by shuffling hubs randomly over the entire brain (10,000 permutations).

### Comparison of hubs with previous findings

Previous work has delineated a fear-anxiety-circuit in the human brain^40,41^. This circuit has been derived meta-analytically by combining neuroimaging studies on social anxiety disorders (SAD). Brain regions implicated in the proposed fear-anxiety circuit are those regions that show significant activation increases in SAD patients compared to healthy controls. We identified 40 regions in our whole-brain partition that were reported in the most recent meta-analysis on the fear-anxiety circuit based on a mapping of Brodmann areas^31^. We quantified the spatial overlap between these regions and the combined in-out-connectivity change hubs from the present analysis with the Jakkard index^42^ and odds ratio. Significance of the Jakkard index was assessed based on 10,000 random permutations of hubs across the brain. Significance of odds ratio was assessed based on the 95%-confidence interval.

We also compared the topology of hubs to the association map for *fearful faces* from Neurosynth (Neurosynth.org). Neurosynth is a publicly accessible database that currently lists the results from >14,000 functional MRI investigations for automated meta-analyses and probes the association between cognitive terms and brain activation^43^. Neurosynth’s association map for fearful faces is based on 91 functional neuroimaging experiments. It contains voxel-level z-scores that contain information on the specificity between a term (here: fearful faces) and a brain region. We averaged z-scores within each node of the Shen partition. Permutation tests with 10,000 permutations were used to assess whether hubs and non-hubs differed in association strength with the term *fearful faces*, both across the entire brain and within different ICN. For this test, significance was assumed when the empirical difference between hubs and non-hubs was larger than the difference in 95% of permutations.

### Ethics Statement

We obtained permission from the Human Connectome Project to work with the open access and restricted access data. Informed written consent was obtained from all participants^30^. We further obtained approval from the Institutional Ethics Board at the University of Bonn, Germany. All methods were performed in accordance with the relevant guidelines and regulations.

## Results

We analyzed resting-state and affective processing data from N = 843 healthy participants enrolled in the Human Connectome Project^30^. All analyses were based on a whole brain parcellation scheme that ensures regions of interest with a high degree of functional homogeneity that are highly consistent and reproducible across participants^44^.

### Intrinsic connectivity networks

**Figure 1:**
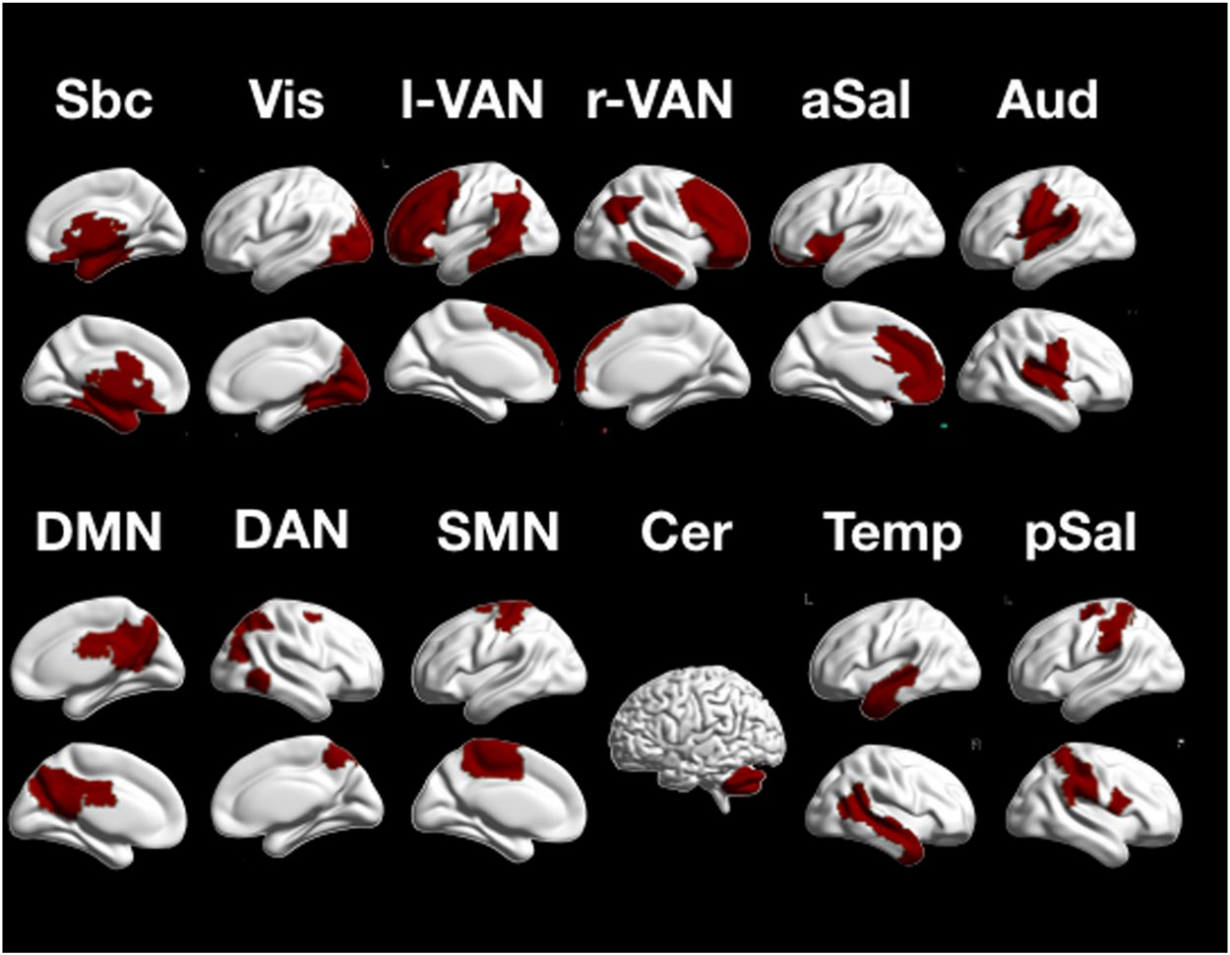
Twelve ICN obtained by group-consensus clustering with the infomap algorithm: Sbc - subcortical network, Vis - visual network, l-VAN - left ventral attention network, r-VAN - right ventral attention network, aSal - anterior salience network, Aud - auditory network, DMN - default mode network, DAN - dorsal attention network, SMN - somatomotor network, Cer - cerebellar network, Temp - temporal network, pSal - posterior salience network.

Our first analysis was intended to delineate ICNs in the Shen parcellation. This was achieved by applying a consensus clustering approach with the Infomap module detection algorithm to functional connectivity matrices based on partial correlations^45^. We identified twelve ICNs at the group level that correspond well to the previous literature (see Fig. 1). ICNs include a large subcortical network (54 ROIs), a visual network (33 ROIs), a right (31) and left (25) lateralized ventral attention network, an anterior salience network (26) ROIs, a superior temporal network (20), a posterior default mode network (14), a dorsal attention network (15), as well as a somatomotor (17), cerebellar (14), medial temporal (13), and posterior salience network (11 ROIs). We did not find a separable executive control network. The two ICNs that we labeled “ventral attention networks”, however, included larger parts of lateral prefrontal cortex and are presumably involved in executive control.

### Task evoked activations

Our first research question asked for brain activation evoked by an affective processing task and how activation topology relates to the topology of ICNs. The experimental paradigm for affective processing utilized a block design in which participants altered between an affective condition that required the matching of face stimuli with expressions of fear and anger and a control condition that required the matching of geometrical shapes. Task evoked activation differences were analyzed using weighted linear contrasts in a general linear model framework.

**Figure 2:**
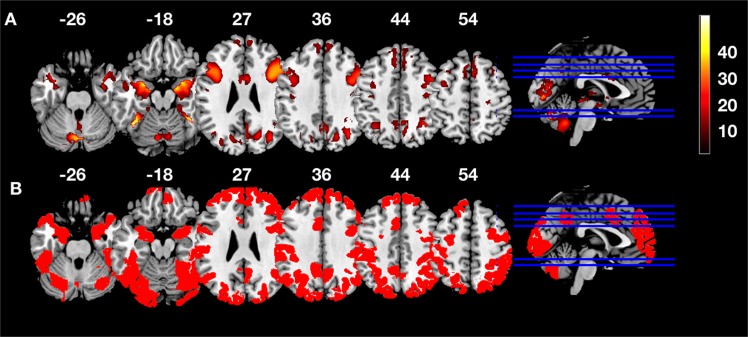
The top row (**A**) shows task evoked activations (t-values) from the contrast affective> neutral (FWE-corrected). The bottom row (**B**) shows brain regions whose connectivity changes qualified them as hubs.

The group level analysis showed robust widespread activations throughout the brain: We observed large bilateral clusters with peaks in the fusiform gyrus and amygdala (k = 16187 voxels), the cerebellum (k = 1274), the middle cingulate (k = 219), and the supplementary motor area (k = 1323). Unilateral clusters were observed in lateral and medial prefrontal cortex (right hemisphere k = 2829, left hemisphere k = 2784), pre- and post central gyri and the insula. The thresholded group activation map is shown in Fig. 2. Coordinates and statistics are given in Table 1.

**Table 1:** Peak activation coordinates and statistics for task-evoked activations from the contrast affective > neutral.

Brain Region	Cluster Size	T	x	y	z	note
fusiform gyrus	16187	47.06	40	−52	−18	bilateral
amygdala		46.86	18	−4	−16	
left cerebellum	1274	31.33	−2	−74	−26	bilateral
right MFG	2829	30.18	38	12	28	
left MFG	2784	26.25	−42	16	28	
middle cingulate	219	19.60	6	6	28	bilateral
right SMA	1323	14.16	4	16	54	bilateral
right precentral gyrus	96	10.68	38	−14	46	
right precentral gyrus	68	10.50	28	−26	60	
right MFG	25	9.80	4	−28	58	
left post central	127	9.19	−46	−12	30	
left insula	82	8.90	−38	−26	14	
right MFG	111	8.57	34	18	54	
left MFG	30	8.55	−4	−24	58	
left MFG	62	7.42	−28	18	54	
right Insula	28	6.52	32	−24	18	

**Figure 3:**
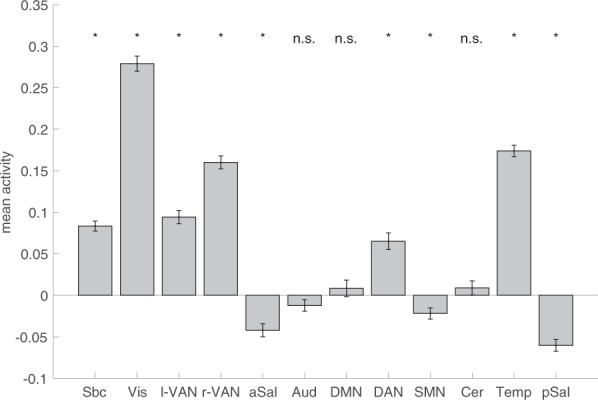
Mean activation of ICN during affective processing. The bars depict means, the error bars depict standard errors of mean. ICN for which mean activity differs significantly from zero are flagged with an asterisk (Bonferroni corrected for twelve comparisons).

We extracted mean parameters for each ICN and from each participant to assess whether the ICN responded to the task. Results are shown in Fig. 3. All ICNs except for the auditory network, the default mode network, and the cerebellar network responded to the task (i.e. showed a clear increase or decrease in mean activity between task conditions). The subcortical, visual, temporal, and attention network increased their activity during the processing of affective information. The two salience networks as well as somatomotor network decreased their activity.

### Within and between network connectivity

**Figure 4:**
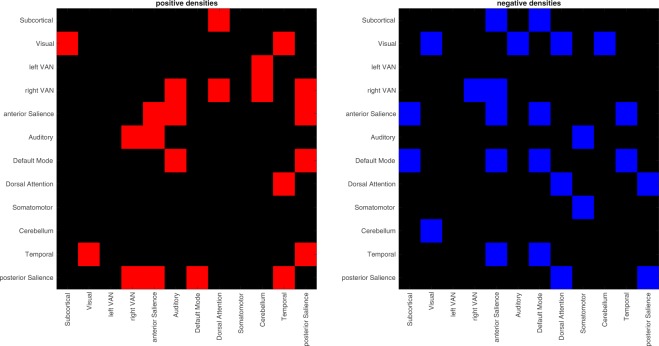
Changes in connection density within and between ICN. Red indicates increases in connectivity during affective processing, blue indicates decreases. Within network densities are plotted on the diagonal. Please note that the matrices are not symmetrical due to the directed nature of PPI analyses.

Our second research question asked for task-evoked connectivity changes and how these connectivity changes fall into different ICNs. We quantified task-evoked changes of functional connectivity within and between ICNs as the proportion of edges that changed connectivity relative to the total number of edges (i.e. density) within each ICN and between any pair of ICNs. All calculations were based on the difference matrix, i.e. a directed and signed matrix where edges that increased their connectivity in response to the task were flagged with 1 and edges that decreased their connectivity with −1 (see methods). Significance of the density measures was assessed based on 10,000 permutations of the difference matrix. Results are shown in Fig. 4.

**Figure 5:**
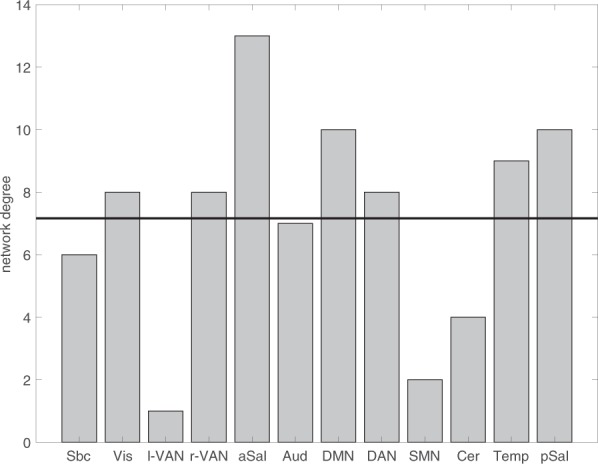
Network-level degree for all twelve networks. The black horizontal line indicates the hypothetical degree under the assumption that all densities distribute equally across networks.

We characterized each ICN by its network-level degree (Fig. 5). Each ICN had a network-level degree >1, indicating that each ICN responded to the task. The highest degrees (> =10) were observed for the anterior salience network, the default mode network, and the posterior salience network. The lowest degree was observed for the left ventral attention network. Despite this marked descriptive differences, the deviation from a uniform distribution was barely non-significant (χ^2^ (11) = 18.372, *p* = 0.073).

### Nodal change during affective processing

**Figure 6:**
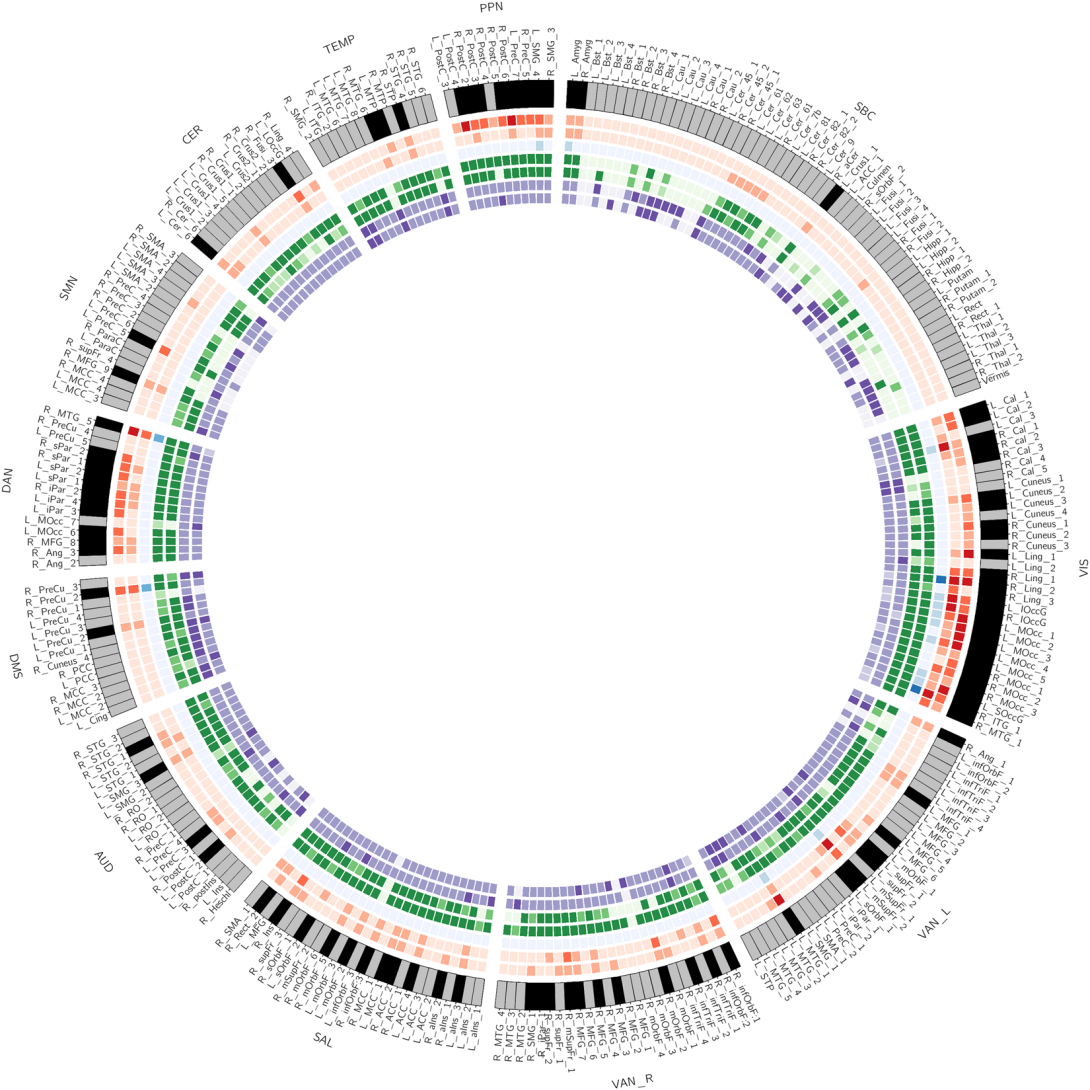
Connectivity change hubs during affective processing. The plot shows all brain regions ordered according to their network allegiance. Hubs are flagged in black in the outer layer. The heat maps visualize the different centrality measures (from outwards to inwards): in and out degree (red), betweenness (blue), in and out participation coefficient (green), and in and out path length (purple). All values are z-standardized for display purposes.

**Figure 7:**
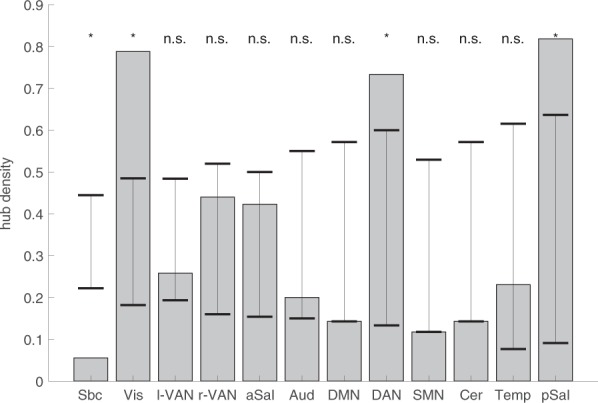
Hub density per network (number of hubs relative to nodes per ICN). Hubs were derived based on hub-scores (see methods). The black lines indicate significance thresholds based on 10,000 permutations. Densities outside this range are considered significant (two-sided) and are marked with an asterisk.

Our third research question asked for connectivity changes at the level of brain regions, and how this relates to different ICNs. We calculated nodal degree, betweenness centrality, the participation coefficient, and nodal path length for each node based on the difference matrix. For this analysis, the difference matrix was converted into an unweighted directed graph with only positive edges (see methods). Based on the four metrics, we calculated hub-ness based on hub-scores^37^. A brain region was treated as hub when the region scored in the top 33% on at least three out of four centrality measures. As an alternative way to identify hubs based on centrality measures we applied k-means clustering to the four centrality measures (see methods). Please note that our definition of hubs is based on the difference matrix and reflects the hub-ness of task-evoked connectivity changes. In the present context, hubs represent brain regions whose connectivity pattern suggests a particular strong modulation through the task and a central and important role within the task-evoked network. Of the 273 nodes in our whole brain partition, the connectivity changes of 92 nodes (93 nodes based on k-means) were strong enough across the four centrality metrics to qualify as hubs. The two methods yielded very consistent results. 85 brain regions were identified as hubs with either method. Given this consistency and that hub-scores is the more widely applied method, we decided to display only the results based on the more common used hub-scores in Figs. 1b and 6. Hubs were responsible for most connectivity changes throughout the connectome. From the total of 7742 between-node connectivity changes, 3668 (k-means: 3844) occurred between hubs and 3643 (k-means: 3441) between hubs and the periphery. Only 431 (k-means: 457) edges changed their connectivity between non-hubs. As can be seen from Fig. 7, hubs were located in all twelve ICNs, albeit with marked differences in densities. We noticed that hubs were particularly pronounced in the visual network (*p* < 0.001, Bonferroni corrected), dorsal attention (*p* = 0.02, corrected), and posterior salience network (*p* = 0.017, corrected). In these three networks, the proportion of hubs was higher than expected by chance. Within the subcortical network, the number of hubs was significantly lower than expected by chance (*p* < 0.001, corrected). This is particularly noteworthy because subcortical areas have traditionally been implicated in affective processing: The left and right amygdala stood out as two out of three hubs in the subcortical network.

### Comparison with published results

Our last question asked how task-evoked changes in functional connectivity during affective processing in healthy participants relate to structural and functional alterations observed in patient populations. Previous work has delineated a fear-anxiety-circuit in the human brain^31,40^. This circuit has been derived meta-analytically by combining neuroimaging studies on social anxiety disorders (SAD). Brain regions implicated in the proposed fear-anxiety circuit are those regions that show significant activation increases in SAD patients compared to healthy controls. We identified 40 regions in our whole-brain partition that were reported in the most recent meta-analysis on the fear-anxiety circuit^31^ We quantified the spatial overlap between these regions and the combined in-out-connectivity change hubs from the present analysis with the Jakkard index which standardizes the intersection between two groups of nodes with their union^42^. We found a Jakkard index of 0.245 for hubs based on hub-scores and 0.243 for k-means (p < 0.001, permutation testing with 10,000 permutations), indicating substantial overlap between our hubs and the fear-anxiety circuit (with 40 nodes in the fear-anxiety circuit and 92 (93) connectivity-change hubs, the upper limit of the Jakkard index is 0.435 (0.43)). We also computed the odds ratio of a node being a connectivity change-hub when being also part of the fear-anxiety-circuit. The odds ratio was 4.699 (95%-confidence interval: [2.315, 9.35529]) for hub based on hub-scores and 4.601 (95%-confidence interval: [2.267, 9.349]) for hubs based on k-means clustering, which is a further indicator of a substantial relationship between the present results and the fear-anxiety-circuit in SAD.

To compare hub topology with the specificity of activation as documented across different literatures, we obtained the association map for fearful faces from Neurosynth (see Methods). While we did not find higher association strength for hub regions than non-hubs at the whole-brain level (empirical difference: 0.071, *p* = 0.0977, permutation test), we did find such difference when only focusing on the subcortical network (empirical difference: 2.519, *p* = 0.0096, FWE-corrected for twelve tests, permutation test). No difference in association strength survived Bonferroni-correction in any other network.

## Discussion

The aim of the present investigation was to map changes in neural activation and functional connectivity evoked by an affective processing task and relate these changes to the topology of intrinsic connectivity networks. Wide-spread changes in activity and connectivity implicated all ICNs in affective processing but also indicated some selectivity in subcortical regions, particularly the amygdala.

We first delineated ICNs in the resting-brain using graph-analytical methods. This data-driven approach resulted in twelve ICNs that are at large consistent with those reported in the literature: The visual, salience, default mode, somatomotor, auditory, and ventral and dorsal attention belong to the canonical ICNs^10,11^. The distinction between ventral and dorsal attention systems has been documented in the literature^46^ as well as the distinction between the anterior and posterior salience network^47^. We did not find a clear control network, however, the ventral attention networks included large parts of lateral prefrontal cortex and thus also resemble the fronto-parietal control network^48^. Our partition subsumed subcortical regions to one large subcortical network. This finding may seem unusual because subcortical regions are often assigned to the salience network. Some studies, however, have found evidence for a separable network that includes the basal ganglia and associated subcortical nuclei^49,50^. Furthermore, separate subcortical communities are not uncommon in graph-theory based partitions^35^ and one study that applied graph-theoretical community detection within the same whole-brain parcellation has found a separable subcortical ICN like we did^45^. It could be the case that intrinsic properties of subcortical signals have contributed to the size of the subcortical ICN. Functional connections of subcortical brain regions are generally less reliable when compared to cortical regions^51^. This effect, on the other hand, can be ameliorated by longer acquisition times (as applied in the present investigation). Further, reliability seems to be particularly low within the cerebellum which we classified as its own distinct ICN. It is therefore unlikely that the large subcortical ICN is just a byproduct from signal stability. The only ICN not frequently reported in the literature is the temporal network. Given the overall meaningful network partitioning and the symmetry of the temporal network across hemispheres, we still believe that our temporal network is a valid ICN.

Almost all of the ICNs were modulated by the task, either by increasing or decreasing their activity. Consistent activation or deactivation of brain regions within ICNs is a core feature of brain organization and is in line with notions that (a) the topology of ICNs resembles the topology of task-evoked activation^11,52^, that (b) ICNs are predictive of task-evoked activations^12^, and (c) that the brain’s intrinsic modular architecture persists into task states^13^. ICNs that increased their activity during affective processing were mainly those that encompass brain regions devoted to visual processing (visual and temporal network), and those devoted to attention (ventral and dorsal attention networks). An increase in activity was also observed in the subcortical network. Decreased activity was observed in the anterior and posterior salience network as well as in the somatomotor network. The default mode network as the prototypical task-negative network did not decrease its activity. This, however, is not necessarily surprising because the most pronounced task-negative behavior of the DMN is usually observed during challenging and attention-demanding tasks^53–55^. A rather surprising finding is the deactivation of the two salience networks. The salience network responds - as the name readily suggests - to salience that should be higher during the processing of affective information^7,56^. Furthermore, the salience network has been repeatedly associated with anxiety and other affective states^15,16,57,58^. While we did not find evidence for an increase in activity of both salience networks, we found them to be most relevant for within and between-network connectivity changes, which is again consistent with its alleged role in affective processing. A closer examination of the activation map revealed that two components of the anterior salience network were differently modulated by the task: The insula regions increased their activity, the anterior cingulate, however, decreased its activity. Such opposite activation pattern within the network has been observed before and qualifies the apparent deactivation of the network as a whole^59^.

Connectivity changes during the task were estimated with gPPI. This allowed us to directly contrast functional connectivity during affective processing with functional connectivity in the control condition. Around 10% of all possible connections in our brain parcellation responded significantly to the task after stringent control for the family wise error. Connectivity changes occurred throughout the functional connectome and implicated all twelve ICNs in affective processing. The ICN with the most pronounced connectivity changes was the anterior salience network, followed by the posterior salience and default mode network. The anterior salience network showed other interesting properties aside its high network-level degree: It was the only network that showed an increase in its positive within-network density, i.e. increases in functional connectivity occurred within the anterior salience network above chance. It further showed the largest diversity in its between-network densities, connecting to six other networks. Together with the high network-level degree of the posterior salience network, these results confirm the pronounced role of the salience network in affective processing mentioned earlier. It is interesting to note that the three networks with the most frequent between network connectivity changes were all networks that did not seem to respond positively to the task at the activation level. Previous research has shown that task-evoked changes in activity and task-evoked changes in connectivity can be dissociated to a certain degree, and that both level of analysis are necessary to fully map a brain region’s response to a task^60^. We conclude that networks related to visual processing and attention respond to visually presented emotional information by increasing their activation while networks related to salience processing and self-referential thought respond mainly by changing their connectivity patterns.

We also investigated the topology of network hubs. Network hubs have received much attention in connectome research^61,62^ because of their important role for integrative processing^63^ and their relevance for disease states^64,65^. Hub regions are central in the connectome, route information exchange, and achieve integration of otherwise segregated processes^62,66^. Previous work has studied the role of network hubs in cognitive processing by first identifying hubs in structural networks or functional resting state networks and then studying their involvement during task states^45,67^. We took a different approach and calculated hubs from the difference matrix: Hubs were thus defined based on the degree, diversity, and centrality of their connectivity changes during the task. We identified more than 90 of such “connectivity-change” hubs that were distributed across all twelve ICNs and were involved in the vast majority of connectivity changes throughout the connectome. Even though the topology of hubs again confirmed that all ICNs are involved in affective processing, four ICNs stood out by having a hub density above (posterior salience, dorsal attention, and visual network) or below chance level (subcortical network). Even though the subcortical ICN was the largest ICN in terms of brain regions (54), it encompassed only three hubs, including the left and right amygdala. This indicates a high level of regional specificity of connectivity changes in subcortical parts of the connectome, a finding that is in line with the high specificity of brain activation changes in the amygdala during the processing of fearful faces, which we were able to further confirm by interrogating the Neurosynth data bank.

One explicit goal of behavioral neuroscience is to explain mental faculties such as emotion and cognition as functions of the brain. Traditionally, the lead paradigm has followed a localizationist approach that presumes that mental faculties can be precisely localized in distinct brain regions or in distinct circuitry of several brain regions. This idea is central to primary emotion theory. This theory presumes that emotions form distinct categories (such as fear, anger, sadness, happiness, disgust, surprise) are not only shared among humans but are evolutionary preserved across mammalian species. This implies a genetic foundation for distinct emotional categories and in consequence also biologically hardwired circuitries for each emotional category in the brain. Empirical support for the localizationist assumption comes from studies in non-human mammals that study the effect of experimental lesions and electrical brain stimulations on the behavioral expressions of different emotional categories. The main finding here is that even the most severe cortical lesions do not affect the expression of emotions but that localized electrical stimulation of subcortical nuclei produces behavioral expressions of emotion that differ depending on the stimulation site^24^. More recently, however, a systems neuroscience account has gained popularity that explicitly positions itself against the localization of emotions in circumscribed brain regions or systems. The proposition builds in part upon the large body of neuroimaging research on ICNs. ICNs are though to support basic processes that together give rise to cognition and emotion^19^. According to this view, there are no circumscribed systems for distinct emotional categories. Emotions and other mental faculties arise from the interplay of ICNs that themselves perform basic routines and represent the building blocks of the mind. Support for this view of constructed emotions comes from neuroimaging work that could not establish correspondence between different emotion categories and ICNs^16^ and re-interpretations of most previous research from a constructivist point of view^68^. In the present study, we capitalized on a task where participants paid attention to emotionally salient facial expressions (prototypically fearful and prototypically angry faces). Even though the present paradigm was not designed to explicitly test primary emotion theory and the theory of constructed emotion against each other, we can still derive important information on the validity of these two accounts. First, we observed that all intrinsic systems of the brain responded to the task, i.e. changed their activity level or their connectivity when viewing emotional faces as compared to the control condition. We found little evidence for any clear leading role of any of the ICNs. This is not only in line with previous findings on the role of ICNs in affective processing but is also in line with the systems level interpretation of the theory of constructed emotion^16^. On the other hand, we did observe a certain degree of specificity in subcortical regions. According to primary emotion theory, brain regions in the subcortical ICN would be candidate regions for the present task. Of these regions only three showed hub-like properties, including the bilateral amygdala. We also interrogated the Neurosynth database to obtain a map of brain regions that respond to fearful faces with high specificity and are thus most likely to be directly involved in their processing. We found that hubs overlapped strongly with this map, but only in the subcortical network and not in any other network. The amygdala is regarded the key region for negative emotions, particularly fear^69^, but also anger^70^. In sum, our present findings are in line with the theory of constructed emotions as we found a widespread involvement of brain regions and systems in the processing of emotional faces. On the other hand, however, we also found a clear involvement of the amygdala which is in support of localizationalist ideas. But while primary emotion theory assumes that emotional processing can be precisely localized in distinct brain regions, it also assumes that distinct brain regions process different emotions. In this regard, the Hariri task is a limitation because it uses both prototypically fearful and angry faces together in presentation blocks and thus mixes salient negative emotions together. It is thus not possible to disambiguate neural signals to either emotional category which limits the theoretical significance of the present finding for primary emotion theory (but not the theory of constructed emotions). Previous research has for instance shown that the amygdala is rather modulated by emotional salience than by emotional category^71^, which might be a better explanation for the present finding. On the other hand, however, the amygdala is thought to be involved in both the fear and the anger/rage circuit^70^. From this perspective, our results do not necessarily contradict primary emotion theory. Future work needs to assess the connectome-level specificity of emotional reactions in a more controlled way. This should particularly include attempts to disentangle different emotional circuity for distinct emotional categories in order to derive a decisive conclusion on the primary emotion theory. Multivariate pattern detection approaches are particularly promising towards this end. A very recent preprint suggests that different induced primary emotions can be decoded from brain-wide functional connectivity data (in line with primary emotion theory) but that a distributed set of within- and between-network connections support each emotional state (contradicting clear localizationist views)^72^. It might very well be the case that emotions are both integrated and segregated at the same time. A unification of primary emotion theory and the theory of constructed emotions has been deemed desirable before^73^. We hope that we provide a first empirical basis to do so in the future. Another important aspect for future work will be to disentangle emotional observation from emotional experience. While there is evidence the the observation of emotions in others leads to comparable affective states across observers^74^, it would be desirable to also utilize task paradigms that more directly induce emotional states during the experiment.

We also need to mention another limitation of the present study. Next to using both prototypically fearful and angry faces together in presentation blocks, the control condition just required judgments about geometrical shapes. In this regard, it is also not possible to disentangle emotional processing from the processing of faces. We still decided to utilize the emotional task data from the HCP as the task protocol is one of the most widely studied paradigms in the field that elicits robust hemodynamic reactions^75^ and because it gave us the unique opportunity to include a very large number of participants (i.e. N = 820). Future work, however, will need to address the disentanglement of the emotional from the face connectome^76^. The large number of participants made it possible to control the family-wise error at the connection level while still retaining enough statistical power to detect wide-spread and meaningful changes throughout the connectome. While task-based connectomics and a cognitive network neuroscience have been gaining momentum in recent years^77^, there is still no gold standard in how to best derive and utilize task-connectome-matrices. We here propose to use binary difference matrices that reflect task-induced connectivity changes for further network analysis. By using a difference matrix we derived several novel insights into the reorganization of the functional connectome when processing emotional faces: the involvement and interactions of all ICNs and the topology of connectivity-change hubs that are over-abundant in the visual, dorsal attention, and salience systems and highly specific within the sub-cortical network where they correspond to those regions that are not only sensitive but also specific to the processing of emotional faces. Through the use of spatial permutation-based testing on the difference matrix for our main research questions, we also mitigated problems that might have arisen from spurious connections due to the large sample size. We hope to encourage future investigations with this approach and believe that our results are of interest for different theoretical accounts of the neurobiology of emotions.

Research into affective processes and their representation in the brain are also relevant from a clinical perspective^78^. Affective disorders belong to the most prevalent psychiatric disorders and constitute a major public health burden^79,80^. Clinical research has derived a list of brain regions that differ functionally between patients suffering from anxiety disorders and healthy control participants^31,41^. This fear-anxiety circuit has been derived meta-analytically by applying activation likelihood estimation^81^ and thus includes regions that show consistent alterations in their functional activity properties in patients. The fear-anxiety circuit overlaps with brain regions whose connectivity changes during affective processing suggest hub like properties. This validates the fear-anxiety circuit by confirming that implicated brain regions form the basis of emotional processing in healthy participants and are thus likely to constitute the neural basis of the brain’s emotional response. Resting-state connectivity studies also seem to support the notion of a fear-anxiety circuit in patients^31^, however, studies are still inconsistent and to our knowledge no method has been proposed as yet to combine single connectivity results in an unbiased way through meta-analysis. Once such approach will become available it will be important to extend our present comparison from a nodal (i.e. brain region) to a connection-level perspective. Future work might also use our results as a starting point to obtain a more algorithmic view of affective processing on the brain level and obtain evidence for more biologically informed interventions for anxiety disorders. It also needs to be mentioned that the term fear-anxiety circuit is not without criticism. A long line of animal research suggests that fear and anxiety are actually separable emotions that rely on their respective neural circuitry^82–84^. These circuits, however, show a certain degree of overlap, particularly with regard to the amygdala. Recent optogenetic work has demonstrated that different amygdala sub nuclei play different and sometimes even opposing roles in fear and anxiety (see ref. ^84^ for review). From this perspective, it is a clear shortcoming that we treated the entire amygdala as a single network node and did not attempt to dissociate the network-level contributions from different nuclei. While human MRI protocols are able to detect dissociable signals from amygdala sub nuclei^85,86^, it is only now that available methods approach a sufficient resolution level to visualize most nuclei in structural scans^87^. Once it becomes possible to disentangle functional connectivity of different amygdala sub-nuclei precisely within the amygdala and between the amygdala and the rest of the brain, steps can be taken to study the functional amygdala network in more detail, both in the resting state and during task states that allow to dissociate fear and anxiety responses in humans^88^.

## Conclusion

We have provided the most comprehensive and detailed mapping of functional connectivity evoked by emotional faces in the largest data set so far. Our results provide arguments for the current debate how emotions are represented in the brain and point towards clinically relevant circuitry within the functional connectome.

## Data Availability

All data are available from the Human Connectome Project (www.humanconnectome.org) upon registration. Data use terms can be accessed via https://www.humanconnectome.org/study/hcp-young-adult/data-use-terms.
